# Perithyroidal Salivary Gland Acinic Cell Carcinoma: Morphological and Molecular Attributes of a Unique Lesion

**DOI:** 10.1007/s12105-020-01187-3

**Published:** 2020-06-09

**Authors:** C. Christofer Juhlin, Sylvia L. Asa, Kenbugul Jatta, Homeyra Naserhojati Rodsari, Ivan Shabo, Felix Haglund, Brett Delahunt, Hemamali Samaratunga, Lars Egevad, Anders Höög, Jan Zedenius

**Affiliations:** 1 Department of Oncology-Pathology, Karolinska Institutet, Stockholm, Sweden; 2 Department of Pathology and Cytology, Karolinska University Hospital, Stockholm, Sweden; 3 University Hospitals Cleveland Medical Center, Case Western Reserve University, Cleveland, Ohio USA; 4 Department of Breast, Endocrine Tumors and Sarcoma, Karolinska University Hospital, Stockholm, Sweden; 5 Department of Molecular Medicine and Surgery, Karolinska Institutet, Stockholm, Sweden; 6 Department of Pathology and Molecular Medicine, University of Otago, Wellington, New Zealand; 7 Department of Molecular and Cellular Pathology, University of Queensland, Brisbane, Australia

**Keywords:** Acinic cell carcinoma, Thyroid, Mutation, Gene fusion

## Abstract

Rarely, salivary gland tumors such as mucoepidermoid carcinoma, mammary analogue secretory carcinoma and mucinous carcinoma arise as primary tumors from ectopic or metaplastic salivary gland tissue adjacent to or within the thyroid gland. We report for the first time a case of primary salivary acinic cell carcinoma (AcCC) adjacent to the thyroid gland in a 71-year-old female patient with Crohns disease and a previous history of malignant melanoma. Following the development of a nodule adjacent to the left thyroid lobe, a fine-needle aspiration biopsy was reported as consistent with a follicular lesion of undetermined significance (Bethesda III). A left-sided hemithyroidectomy was performed. A circumscribed lesion measuring 33 mm was noted adjacent to the thyroid and trapping parathyroid, it was composed of solid nests and glands with microcystic and follicular patterns. The tumor was negative for thyroid, parathyroid and paraganglioma markers, but positive for pan-cytokeratins, CK7, CD10, CD117, androgen receptor and HNF-beta. A metastasis of a thyroid-like renal cell carcinoma was suspected but ruled out, and the patient had no evident lesions on extensive radiology of the urogenital, pulmonary and GI tracts. Based on the morphology, a diagnosis of AcCC was suggested, and confirmed with DOG1 and PAS-diastase staining. Molecular analyses pinpointed a constitutional *ASXL1* variant of uncertain significance, but no fusion events. The patient had no radiological or clinical evidence of parotid, submandibular or sublingual tumors postoperatively, and the excised lesion was therefore assumed to be a primary tumor. We here detail the morphological and immunophenotypic profile of this previously undescribed perithyroidal tumor.

## Introduction

Although the bulk of thyroid tumors are derived from the follicular epithelium, a number of unusual lesions from stromal or ectopic tissue components have been reported, including angiosarcoma, parathyroid tumors, paraganglioma, peripheral nerve sheath tumors, solitary fibrous tumors and thymomas, to name a few [1]. Moreover, rare cases of salivary gland tumors have also been described within or adjacent to the thyroid gland, including mucoepidermoid carcinoma, sclerosing mucoepidermoid carcinoma with eosinophilia, mammary analogue secretory carcinoma and mucinous carcinoma [1–7]. These malignant epithelial neoplasms are thought to arise in ectopic salivary gland tissue and are histologically identical to those arising in salivary glands, but often display focal expression of thyroid-related markers such as TTF1, PAX8 and thyroglobulin [1]. From a prognostic perspective, these tumors are often associated with poor patient outcome due to the development of regional and distant metastases [1, 8].

Acinic cell carcinoma (AcCC) is a low-grade malignant neoplasm that almost exclusively occurs in the major salivary glands, especially the parotid gland, and is usually diagnosed on cytological examination following a fine needle aspiration biopsy (FNAB) [9]. Besides being primarily known as a salivary gland tumor, AcCC also occurs in the pancreas where it derives from pancreatic acinar cells. Salivary-type AcCCs have also been described in the lung, stomach, prostate and breast [9–13]. The tumor is predominantly found in women, and the usual age at diagnosis is > 50 years [9, 14]. Previous radiation exposure has been described as a risk factor, and a hereditary link has also been suggested for small subsets of cases [15, 16]. Although slow growing, AcCCs carry potential for metastatic spread, and long-term follow-up is recommended for most tumors following surgical excision. Histologically, AcCCs usually display a solid, micro-cystic, papillary or follicular growth pattern, and often display immunoreactivity for broad-spectrum keratins, DOG1 and CD117 (c-Kit) [17–22]. Moreover, cytoplasmic droplets of mucin visualized through a Periodic acid-Shiff stain with diastase (PAS-D) have also been described as diagnostic [18, 23].

Given the rarity of AcCC, the underlying genetics driving the development of these tumors have only partly been deciphered. Distinct cytogenetic aberrancies have been described, including deletions covering the short arm of chromosome 6 [24]. Moreover, dysregulation of Rb-mediated growth suppression has also been suggested as a contributing event [25]. To add on this, a recent study observed *HTN3-MSANTD3* fusions in a subset of salivary gland AcCCs with an indolent clinical course [26].

In this report, we report a case of AcCC clinically presenting as a thyroid mass, and portray the clinical, histological, immunohistochemical and molecular landscape of this rather unique manifestation.

## Case report

**Fig. 1:**
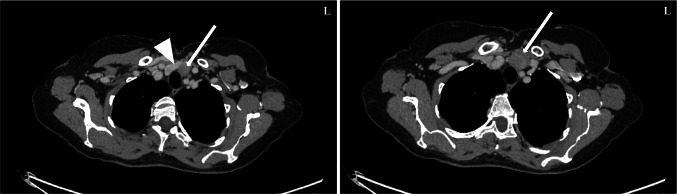
Preoperative computerized tomography (CT) scan of the superior thoracic inlet. The thyroid gland is marked by a white arrowhead, and the white arrow designates the adjacent 24 mm lesion originally believed to constitute an enlarged perithyroidal lymph node in level VI. The image on the right is taken caudal to that on the left and highlights the maximum diameter of the mass, reaching the superior thoracic aperture

**Fig. 2:**
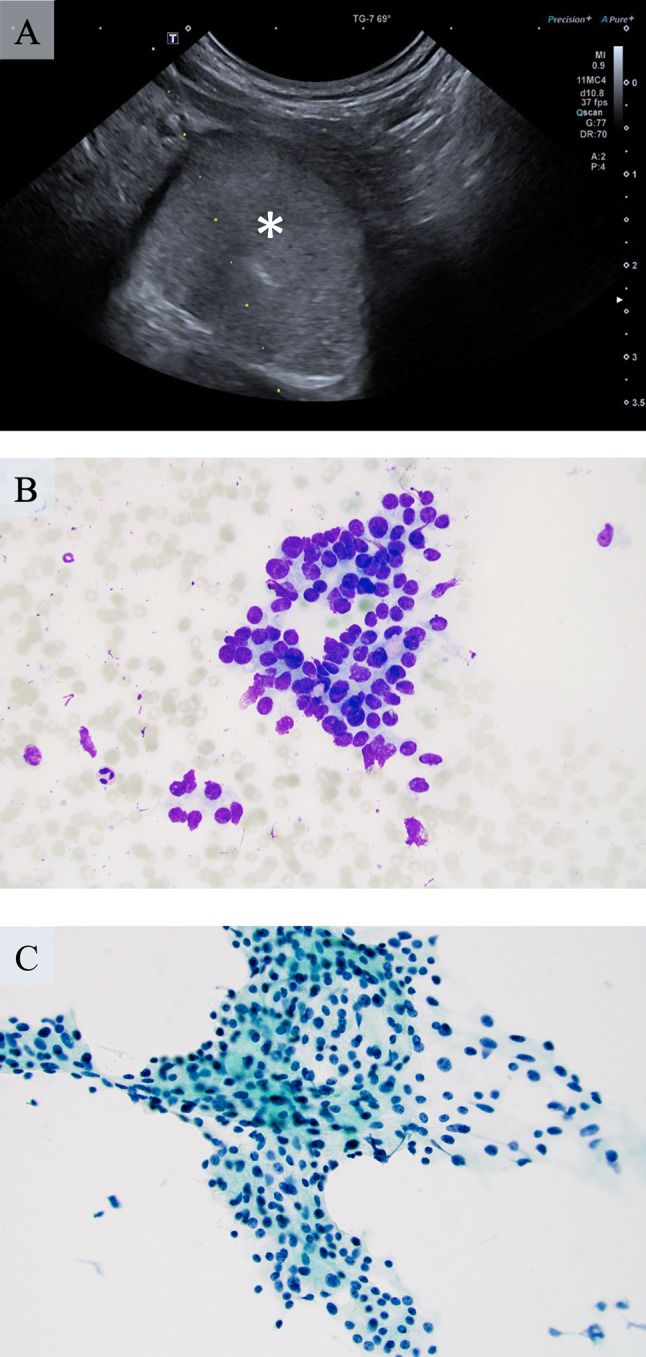
Ultrasonographic and cytologic hallmarks of the acinic cell carcinoma (AcCC). **a** Ultrasonography-guided fine-needle aspiration biopsy (FNAB) of the tumor (white asterisk). Note the appearance of the needle tip in the central part of the lesion (directly underneath the asterisk). **b** May-Grünwald Giemsa (MGG) stain of the aspirated cells at × 400 magnification reveals clusters of tumor cells with round to oval nuclei displaying mild atypia. **c** Papanicolaou (PAP) stained smear of the same tumor aspirate

The patient was a 71-year-old female of Swedish ethnicity. She had a medical history of insulin-treated type 2 diabetes, salazopyrine-treated Crohn’s disease and pelvospondylitis. She was previously administered infliximab, but this was later changed to rituximab. In 2008, she was diagnosed with a R0 resected amelanotic malignant melanoma of the left forearm, Breslow’s depth 3 mm, Clark level IV, with no synchronous metastases. As part of the clinical follow-up procedures, the patient was screened for relapses by various imaging techniques, and in July 2019, a computerized tomography (CT) of the thorax and abdomen displayed an incidentally discovered 24 mm nodule that was assumed to be a lymph node adjacent to the caudal aspect of left thyroid lobe (Fig. 1). Shortly afterwards, the patient was referred to our hospital. An ultrasonography-guided fine needle aspiration biopsy (FNAB) of this mass was performed, and the cytology report described hyperplastic follicular thyroid epithelium in groups of varying sizes, consistent with a follicular lesion of undetermined significance (Bethesda III) (Fig. 2). A separate FNAB of the superior aspect of the lobe was consistent with follicular nodular disease. When the patient was seen at the endocrine surgery department, she was clinically and biochemically euthyroid. A decision was made for a diagnostic left-sided hemithyroidectomy, which was performed two weeks later, in which the mass and thyroid lobe were removed en bloc. The operation time was 50 min, and the procedure was complication-free.

### Histopathological Description

**Fig. 3:**
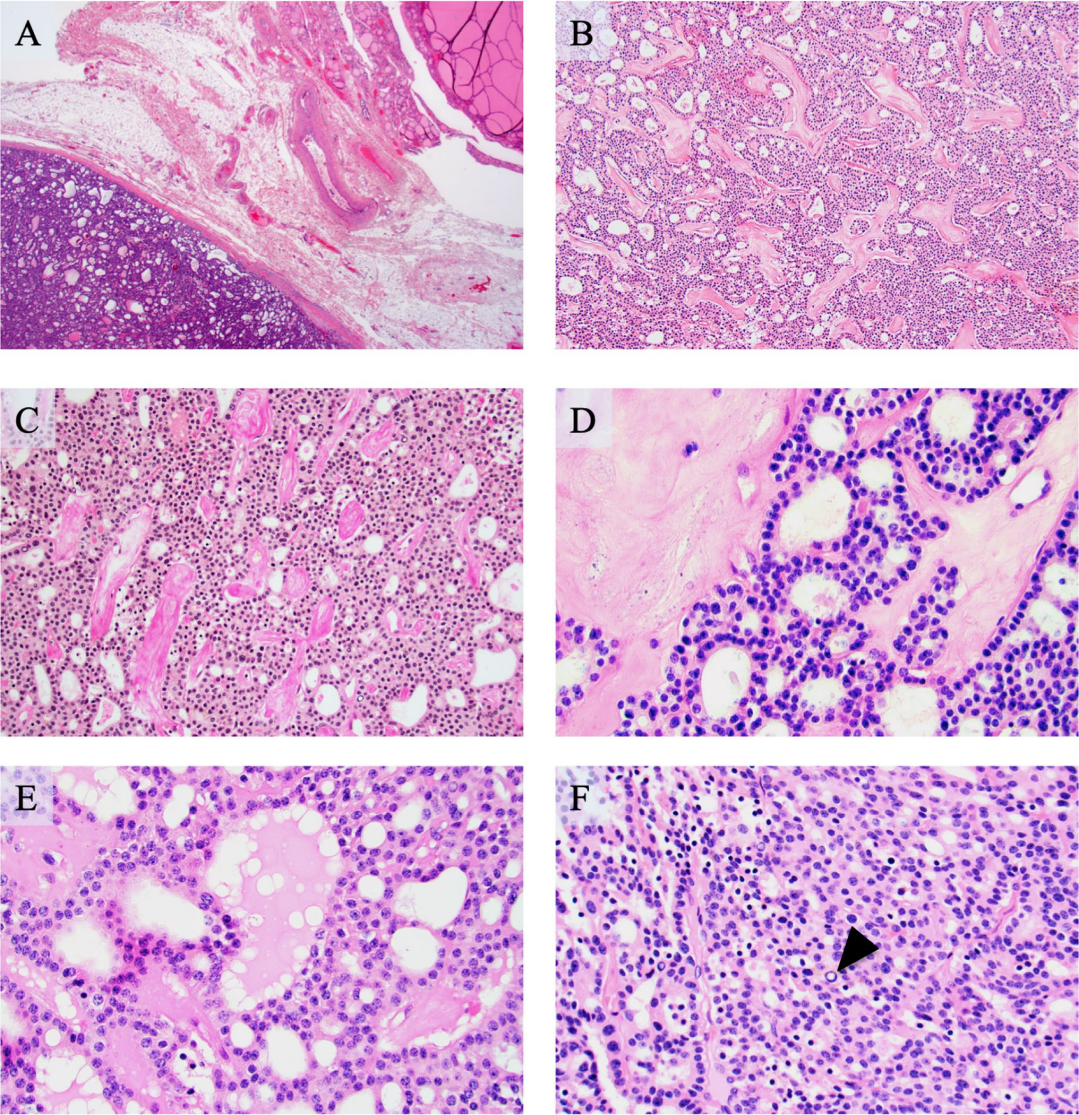
Histological attributes of the acinic cell carcinoma (AcCC). All photomicrographs represent hematoxylin and eosin (H&E) stained tissue sections and are magnified × 100 unless otherwise specified. **a** The AcCC is well delineated and separated from the adjacent thyroid tissue by a fibrous capsule. The thyroid tissue (upper right) displays evidence of follicular nodular disease. There is no evidence of residual ectopic salivary gland tissue in the surrounding tissue. Magnification × 20. **b** The AcCC is composed of solid nests and glands with microcystic and follicular patterns. The tumor cells are large and polygonal, with abundant pale acidophilic granular and vacuolated cytoplasm. The stroma contains irregular and meandering deposits of collagen-like fibrous tissue. **c** Van Gieson (VG) stain at magnification × 200 highlighting the fibrous depositions in the surrounding stroma. **d** High magnification (× 400) view of the micro-cystic growth pattern and surrounding amorphous fibrosis. Note the monotonous appearance of the tumor cells. **e** Area with a microfollicular growth pattern and colloid-like accumulations, mimicking that of a follicular thyroid neoplasm. Magnification × 400. **f** Solid area, with focal findings of nuclear inclusions (arrowhead). Magnification × 400

**Fig. 4:**
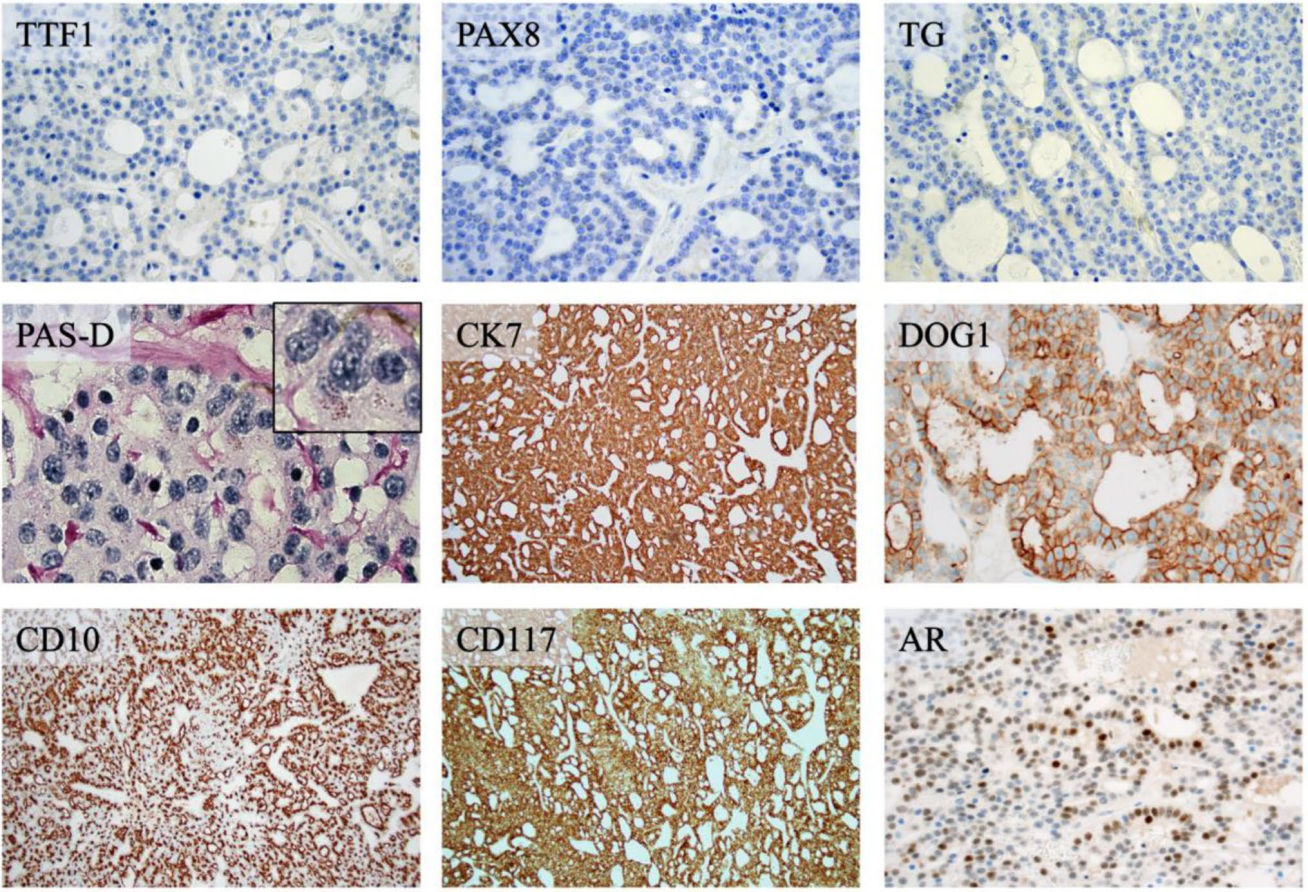
Histo- and immunohistochemical profile of the acinic cell carcinoma (AcCC). *Top row, from left to right*: TTF1, PAX8 and thyroglobulin (TG) stains, all unequivocally negative. *Middle row, from left to right*: PAS diastase (PAS-D) staining displaying intracytoplasmic PAS positive droplets resistant to diastase, thereby verifying them as mucinous. Cytokeratin 7 (CK7) and DOG1 immunoreactivity was evident in the overwhelming majority of tumor cells. *Bottom row, from left to right*: Widespread CD10 and CD117 immunoreactivity, as well as focal androgen receptor (AR) positivity was also evident. Images are magnified × 100 (CK7, CD10, CD117), × 400 (TTF1, PAX8, TG, DOG1, AR) and × 1000 (PAS-D)

The left thyroid lobe with the adherent mass weighed 18.5 g and measured 65 × 30 × 15 mm. In the caudal aspect of the lobe, firmly attached to the thyroid, there was a well circumscribed tumor that measured 33 × 33 × 25 mm and was macroscopically demarcated from the adjacent thyroid tissue and displayed a pale yellow, homogenous cut surface. The thyroid lobe exhibited a variegated cut surface suggestive of follicular nodular disease with hemorrhage. Microscopically, the well-delineated, partially encapsulated tumor was separate from the adjacent thyroid tissue (Fig. 3). Focally, normal parathyroid tissue was seen at the tumor periphery entrapped in the tumor capsule. The tumor was composed of solid nests and glandular formations with micro-cystic and follicular patterns, and the tumor cells displayed monomorphic nuclei and abundant eosinophilic cytoplasm, which was granulated and vacuolated (Fig. 3). A PAS diastase (PAS-D) stain confirmed the presence of small cytoplasmic PAS-D positive droplets (Fig. 4), which were not evident by routine hematoxylin and eosin (H&E) staining.

Immunohistochemical analyses (Fig. 4) confirmed the tumor as derived from non-thyroidal tissues, as no immunoreactivity was noted for TTF1, PAX8, thyroglobulin, chromogranin A, calcitonin and CEA. Parathyroid differentiation was also excluded, as the tumor was negative for PTH and GATA3, while retained immunoreactivity was noted within the surrounding parathyroid cells. The tumor stained for pan-cytokeratins (CKMNF) and CK7, but not CK20, and there was focal EMA positivity. Synaptophysin and AFP yielded equivocal immunoreactivity. Negativity was noted for Hepatocyte-specific antigen, CDX2, MUC1, MUC4, CD31, CD56, inhibin alpha and calretinin. A stain for P53 was consistent with wildtype expression. The Ki67 proliferation index was 1.9%.

Given the history of malignant melanoma, stains for this entity were performed, but consistently negative (HMB45, Melan A, SOX10 and S100). Additional markers were ordered, and although the tumor was positive for CD10, CD117, HNF-beta and vimentin, a diagnosis of a thyroid-like follicular carcinoma of the kidney was ruled out based on the overall morphology and a negative PAX8 stain.

The possibility of a salivary gland lesion was then evaluated. The morphology was not that of a mucoepidermoid carcinoma, and the diagnosis of mammary analogue secretory carcinoma was excluded by negative staining for GATA3, mammaglobin, and GCDFP15. However, the tumor was positive for membranous DOG1, consistent with acinic cell differentiation. The tumor was also variably positive for ER, PgR and AR. The immunoprofile thus strongly supported a diagnosis of AcCC.

### Postoperative Work-Up

Clinical investigations, including magnetic resonance tomography (MRI) of the skull base and neck were performed, and tumor masses in any of the large salivary glands (parotid, submandibular or sublingual glands) were excluded. Previous radiological examinations of the abdomen (CT and MRI scans) were also reviewed, without any detectable lesions in the parenchymatous organs except for multiple hepatic hemangiomas as well as bilateral renal cysts. The remaining right thyroid lobe was also investigated by FNAB, and the cytology report indicated no tumors or atypia (Bethesda II). At present, the follow-up time is 7.5 months, without signs of relapse, but the patient is closely monitored.

### Focused Next-Generation and Sanger Sequencing of Cancer-Associated Genes

The Oncomine Solid Tumor Panel (Ion Torrent S5, Hi-Q Chef; Thermo Scientific, MA, USA) was initially used to interrogate tumor DNA extracted from formalin-fixated paraffin-embedded (FFPE) tissue from the primary tumor using next-generation sequencing (NGS). The panel interrogated > 1800 cancer-associated mutations within *AKT1*, *ALK*, *BRAF, CTNNB1*, *DDR2, EGFR*, *ERBB2*,* ERBB4*, *FBXW7*, *FGFR1*, *FGFR2*,* FGFR3*,* KRAS*, *NRAS*,* MAP2K1*, *MET, NOTCH1*, *PIK3CA*, *PTEN*, *SMAD4*, *STK11* and *TP53*. In short, we found no cancer-related mutations using this panel, nor did we find any evidence of a *TERT* promoter mutation as analyzed by conventional Sanger sequencing. The NGS and Sanger sequencing technologies are based on methodology used in clinical routine practice at our pathology department, and the methodology has been previously published [27, 28]. Given the initial negative outcome, an extended mutational and fusion gene screening using the Oncomine Childhood Cancer Panel was performed using DNA and RNA from the tumor respectively. From a mutational perspective, this panel includes comprehensive mutational hotspot coverage of 86 cancer-related genes and full exon coverage of 44 additional genes, thereby interrogating 130 genes in total. By this analysis, a missense single nucleotide variant (SNV) was observed in the *Additional sex combs like 1 transcriptional regulator* (*ASXL1*) gene (c.3306G>T, p.E1102D), represented in the Catalogue of Somatic Mutations in Cancer (COSMIC) database as COSM36205 (Table 1). This variant is recurrently reported as a somatic mutation in various hematopoietic disorders such as acute and chronic forms of myeloid leukemia, myelodysplastic syndrome and myelofibrosis, and has also been reported in single carcinomas of the breast and lung [29–37]. By our own in silico analyses using the prediction softwares PolyPhen2 and Mutation Taster 2, the SNV was considered pathogenic (Table 1) [38, 39]. We then ran the same mutational pipeline using DNA from normal thyroid tissue from the same operation and detected the same SNV—arguing for a probable constitutional variant. However, as the variant is uncommonly reported in public repositories such as The Genome Aggregation Database (GnomAD, minor allele frequency of < 1%), we designated this variant as a potential constitutional mutation (Table 1) [40]. True germline mutations in *ASXL1* predispose to the Bohring-Opitz syndrome, a condition that most often is congenital and affects an individual’s growth development [41]. Our patient exhibits neither family history nor a physical phenotype suggesting this condition. The ASXL1 protein is a member of the Polycomb group of proteins, characterized by transcriptional regulation mediated by ligand-bound nuclear hormone receptors [42]. As loss of *ASXL1* expression through mutational inactivation seems to be coupled to a proliferative advantage in functional experiments, *ASXL1* is generally considered a tumor suppressor gene [43]. Consulting the Human Protein Atlas (http://www.proteinatlas.org), the *ASXL1* gene is ubiquitously expressed across human tissues, including the thyroid and salivary glands [44].

**Table 1:** Detailed description of the ASXL1 variant discovered by next-generation sequencing

Gene name	Mutation coordinates	Coding sequence	Protein effect	COSMIC ID	Number of mutated samples in COSMIC*	PolyPhen2 prediction	Mutation Taster 2 prediction	MAF (GnomAD)
*ASXL1*	Chr 20:32436018	c.3306G>T	p.E1102D	COSM36205	Haematopoietic and lymphoid: 20, lung: 3, soft tissue: 1, breast: 1	Possibly damaging (0.78)	Disease causing	0.0095

*catalogue of somatic mutations in cancer

### Comprehensive RNA fusion screening

Salivary gland tumors are commonly driven by specific gene fusion events, not least exemplified by recurrent *MYB–NFIB* fusions in adenoid cystic carcinoma, *CRTC1–MAML2* fusions in mucoepidermoid carcinoma, *EWSR1-ATF1* fusions in clear cell carcinomas, *ETV6–NTRK3* fusions in mammary analogue secretory carcinoma [45–48], as well as the recently described t(4;9)(q13;q31) translocation that upregulates *NR4A3* in acinic cell carcinomas [49, 50]. Therefore, to extend our molecular investigations of this highly unusual tumor type, we employed the Oncomine Childhood Cancer Panel to screen for potential gene fusion events. The assay screens for translocations and fusion events for 97 genes covering more than 1700 fusion isoform variants. After manual inspection of the data, no fusion events in any of the probed genes were detected in the AcCC, and there was no expression of *NR4A3* mRNA.

## Discussion

In order to derive important clinical knowledge from exceedingly unusual tumor types, the scientific community is largely dependent on case descriptions and small case series in which tumor phenotypes and clinical outcomes are compared. In this report, the main clinical, histological, immunohistochemical and molecular findings of a unique case of a perithyroidal AcCC are summarized. To our knowledge, this specific entity has not previously been described, although single cases of ectopic AcCCs developing as primary tumors of the lateral low-neck area have been reported [51]. Given the novelty, our findings could merit further investigations in institutional series of thyroid-derived follicular-patterned lesions with equivocal histology and aberrant immunohistochemical profiles. Indeed, the AcCC described here displayed morphology partially reminiscent of a follicular thyroid neoplasm, with a focal follicular growth pattern and colloid-like central deposits (Fig. 3). However, the tumor was unequivocally negative for thyroid-associated markers such as TTF1, PAX8 and thyroglobulin (Fig. 4), setting it apart from other thyroid-related salivary gland tumors that are not uncommonly positive for one or several of these proteins.

Most parotid AcCCs are defined by acinar cell differentiation, but several histological growth patterns have been described, including the micro-cystic and follicular patterns observed in our tumor [17, 52]. Indeed, manifestations of “thyroid-like” appearances have recently been reported in a pancreatic AcCC [53]. Moreover, as the case described here also exhibited PAS-D positive cytoplasmic globules and an immunohistochemical phenotype mirroring that of the parotid tumor counterpart, we are confident that our diagnosis is appropriate. Intriguingly, the AcCC was surrounded by a thin fibrous capsule with focal depositions of parathyroid tissue, and the possibility of an AcCC metastatic to either the thyroid or parathyroid gland was also considered. However, as clinical and radiological investigations found no signs of a synchronous salivary gland mass, the tumor was therefore assumed to constitute a primary lesion. Whether this AcCC originated from ectopic salivary gland tissue or from metaplasia of a non-related cell type remains obscure, although both of these options have been suggested as plausible explanations for the development of salivary gland tumors arising within or adjacent to the thyroid gland [1].

From a molecular standpoint, we detected a constitutional SNV in the *ASXL1* tumor suppressor gene. This SNV is recurrently reported as a somatic mutation in hematological neoplasia, but to our knowledge, this specific variant has never before been reported as a somatic mutation in salivary gland or thyroid tumors. Although not proven on a functional level, one could assume that the *ASXL1* constitutional variant found in our patient potentially plays a role in tumor development, given its association to other neoplasia as well as the damaging properties of the missense variant. Indeed, the glutamate to aspartate change at residue 1102 could in theory disrupt the bordering functional domain responsible for interaction with the retinoic acid receptor (RAR) [42]. As the ASXL1 protein normally augments the anti-oncogenic signals mediated by RAR, our mutation could therefore in theory potentially lead to increased proliferation [42]. Although constitutional in nature, the global minor allele frequency of this SNV is reported in around 0.5%, which would argue against a single nucleotide polymorphism (SNP) of limited biological significance. This is also supported by our *in silico* predictions, suggesting the variant to be impactful. However, the true significance of our findings is yet to be disclosed, and the possibility of this variant being a passenger germline event without clinical consequences must also be considered.

No fusion gene events were detected using a comprehensive clinical panel. Recent findings of *NR4A3* rearrangements in parotid gland AcCCs have been reported and suggested as a driving genetic event; however, no *NR4A3* fusions or even expression of *NR4A3* mRNA were detected in our sample [49]. Although the vast majority of parotid AcCCs do express NR4A3 by immunohistochemistry, the lack of *NR4A3* mRNA expression in our case does not directly argue against the diagnosis, as little is known regarding the *NR4A3* gene status in non-parotid manifestations of this tumor type [50]. Moreover, given the recent discovery of *HTN3-MSANTD3* fusions in subsets of salivary gland AcCCs, it would be interesting to investigate if this genetic aberration was also present in our case [26]. Unfortunately, the gene is not listed among the > 1700 fusion isoform variants included in our clinical routine panel, and hence no such interrogation could be made. Future investigations using pan-genomic characterization could potentially yield additional insights into the molecular etiology of this exceedingly rare tumor manifestation.

The outcome of our patient is uncertain. When primary in the parotid gland, AcCCs tend to recur locally and spread to regional lymph nodes, lungs and skeleton. In some instances, recurrences might occur some 30 years after original diagnosis, which emphasizes the importance of identifying cases at risk of such late events. Prognostication through conventional histology has proven difficult, but traditional features of malignancy in various tumor types have been reported to be more common in AcCC cases with poor prognosis. Prognostic factors include the occurrence of pleomorphism, frequent mitoses, necrosis and invasive growth patterns, including perineural growth. The tumor described here was histologically indolent, as it was encapsulated and had none of the above-mentioned features, potentially suggesting a more benign clinical course for our patient. Even so, an extensive clinical and radiological follow-up is planned.

## Conclusions

We report the finding of an AcCC adjacent to the thyroid gland, a previously uncharacterized tumor entity exhibiting histological and immunohistochemical features similar to AcCCs arising in salivary glands. As this patient displayed a cancer-associated constitutional mutation in the tumor suppressor *ASXL1*, this finding could indicate an underlying molecular aberrancy worthy of follow-up studies. Conclusively, we advocate that surgical pathologists worldwide should be aware of this highly unusual differential diagnosis when assessing tumors of the thyroid gland.

## Data Availability

The data that support the findings of this study are included within the article itself.
